# Ginsenoside Rg1 Alleviates Lipopolysaccharide-Induced Fibrosis of Endometrial Epithelial Cells in Dairy Cows by Inhibiting Reactive Oxygen Species-Activated NLRP3

**DOI:** 10.3390/ani13233723

**Published:** 2023-12-01

**Authors:** Liangli Song, Linnan Wang, Xiangchen Li, Longfei Xiao

**Affiliations:** 1 College of Animal Science & Technology, Ningxia University, Yinchuan 750021, China; sll2019@nxu.edu.cn (L.S.); 13038992003@163.com (L.W.); lixiangchen199906@163.com (X.L.); 2 Veterinary Science (Traditional Chinese Medicine)—Municipal Laboratory of Beijing, Beijing University of Agriculture, Beijing 102206, China

**Keywords:** endometritis, ginsenoside Rg1, EMT, ROS, NLRP3 inflammasome

## Abstract

**Simple Summary:**

Endometritis in dairy cows refers to a process of local inflammatory reaction in the endometrium due to microbial invasion of the damaged endometrium. The aim of this study was to determine the therapeutic effect of ginsenoside Rg1 on the endometritis-induced fibrosis process. The experimental data from this study indicate that ginsenoside Rg1 is able to alleviate the epithelial–mesenchymal transition (EMT) process induced by the lipopolysaccharides (LPS) of bovine endometrial epithelial cell line (BEND) cells, which is related to the inhibition of ginsenoside Rg1 on reactive oxygen species (ROS) accumulation, thereby restraining NLRP3 inflammatory factors from expression. Experiments on mice showed that ginsenoside Rg1 alleviates endometrial fibrosis in mice. The aforementioned results can serve as powerful preclinical evidence for the potential of ginsenoside Rg1 to become an alternative drug for curing endometrial fibrosis.

**Abstract:**

Abnormal function and the fibrosis of endometrium caused by endometritis in cows may lead to difficult embryo implantation and uterine cavity adhesions. Emerging evidence indicates that ginsenoside Rg1 can effectively resist inflammation and pathological fibrosis in different organs. It is hypothesized that ginsenoside Rg1 may possess the potential to mitigate endometrial fibrosis induced by lipopolysaccharides (LPS) in dairy cows. Herein, a model of LPS-stimulated fibrosis was constructed using bovine endometrial epithelial cell line (BEND) cells and ICR mice. Western blotting was used to detect the protein level, and reactive oxygen species (ROS) content was measured by means of DCFH-DA. The uterine tissue structure was stained with H&E and Masson staining. The murine endometrium was assessed for oxidative stress by detecting the concentration of MDA together with the activity of enzymatic antioxidants SOD and CAT. Ginsenoside Rg1 interfered with NLRP3 activation by reducing ROS generation. After the application of ROS inhibitor NAC and NLRP3 inhibitor MCC950, ginsenoside Rg1 could interfere in the ROS/NLRP3 inflammasome signaling pathway by suppressing the epithelial–mesenchymal transition (EMT) of BEND cells. Our in vivo data showed that ginsenoside Rg1 relieved endometrial fibrosis of the mouse model of LPS-induced endometritis by restraining the ROS/NLRP3 inflammasome signaling pathway. Ginsenoside Rg1 inhibits LPS-induced EMT progression in BEND cells probably by inhibiting the activation of ROS-NLRP3 inflammasome.

## 1. Introduction

Endometritis in dairy cows refers to a process of local inflammatory reaction in the endometrium due to microbial invasion of the damaged endometrium. It can cause fibrosis of the endometrium, damage its structure, affect embryo implantation, and even cause miscarriage, seriously endangering the health and reproductive performance of dairy cows. Numerous studies have shown that chronic inflammation is often accompanied by the occurrence of fibrosis [1]. Epithelial–mesenchymal transition (EMT) has been confirmed as a primary factor for the pathogenesis of fibrosis [2]. The mesenchymal cell markers, N-cadherin and α-smooth muscle actin (α-SMA), are up-regulated, while the epithelial cell marker, E-cadherin, which plays a pivotal role in adhesion together with lateral adherent junctions, is down-regulated during EMT [3].

Previous studies have shown that chronic inflammation can cause the accumulation of reactive oxygen species (ROS) [4,5,6], which is an important characteristic of the inflammatory response as well as a crucial contributor to tissue and organ fibrosis [7,8]. According to prior research, mitochondrial oxidative stress is a trigger to activate NOD-like receptor protein 3 inflammasome (NLRP3) [9], which induces various organ pathological fibrosis, including hepatic [10], renal [11], and cardiac [12]. Apoptosis-associated speck-like proteins containing a caspase recruitment domain (ASC, apoptosis-associated speck-like protein containing a CARD), caspase-1, and NLRP3 are essential constituents of a functional NLRP3 inflammasome. In the case of oxidative stress, pro-caspase-1 together with ASC is recruited by NLRP3 to activate caspase-1, subsequently producing bioactive mature IL-18 and IL-1β through the cleavage of pro-IL-18 plus pro-IL-1β [13,14]. At present, it has been confirmed in various tissues that inhibiting ROS production and NLRP3 activation can improve inflammation-induced fibrosis [15].

As an active component of ginseng, ginsenoside Rg1 plays an important role in resisting oxidation, aging, and inflammation [10]. It has been confirmed that Rg1 can alleviate hepatic [16], renal [17], and myocardial fibrosis processes [18]. However, the mechanism for the anti-fibrotic effect of Rg1 in LPS-induced endometrial fibrosis remains to be elucidated.

Therefore, this study aims to investigate whether Rg1 alleviates LPS-induced endometrial fibrosis via repressing NLRP3 and ROS activation through in vitro cell experiments and in vivo mouse models. The research results can serve as a theoretical reference for curing dairy cows with endometrial fibrosis and conducting relevant prevention.

## 2. Materials and Methods

### 2.1. Animals with Corresponding Processing

The Animal Core Facility of Beijing Vital River Laboratory Technology Co., Ltd. (Beijing, China) provided 40 ICR (Institute of Cancer Research, Beijing, China) mice (7–8 weeks old, female) in total, followed by rearing and screening using an animal house (temperature: 20–25 °C; humidity: 40–70%) under a 12 h/12 h dark/light cycle. In addition, the mice had free access to water plus food throughout the study. All the mice were randomly allocated to 4 groups (10 mice for each group) after adaptation for 1 week, including the LPS group (1 mg/mL), the ginsenoside Rg1 group (purchased from MCE (MedChemExpress, Shanghai, China), purity ≥ 99.91%, 100 mg/mL), the ginsenoside Rg1 + LPS group, and the control group. Specifically, in the model group, 50 µL of LPS was injected into the vagina every two days, while in the control group and the ginsenoside Rg1 group, 50 µL of normal saline was injected into the vagina every two days. The mice in the ginsenoside Rg1 group and ginsenoside Rg1 + LPS group received ginsenoside Rg1 (100 mg/kg) for 6 consecutive days by intraperitoneal injection, while the control group and LPS group were intraperitoneally injected with an equal amount of normal saline for 6 consecutive days.

On the final day of drug administration, day 6, the mice all received general anesthesia induced by intraperitoneal injection of 150 mg/kg pentobarbital sodium, and they were subsequently killed. A part of the resected uterus was immediately cryopreserved (−80 °C) and subjected to bioassays, and histological experiments were conducted on the other part of the uterus fixed with 4% paraformaldehyde.

### 2.2. Cell Culturing and Treatment

Bovine endometrial epithelial cell lines (BEND) provided by Shanghai Hongshun Biotechnology Co., Ltd. were cultivated as per the instructions of the supplier. In short, flasks manufactured by Greiner Bio-One (25 cm^2^, Monroe, NC, USA) were utilized to culture the cells with a DMEM/F12 medium, which contained antibiotics (streptomycin at 50 μg/mL + penicillin G at 50 IU/mL) mixed with 10% FBS (Hyclone Laboratories; Logan, UT, USA), followed by placing at 37 °C in a humid environment (5% CO_2_ and 95% O_2_). Once the confluence of 80% was reached, 6-well plates were used for cell inoculation (approximately 1.5 × 10^5^ cells/well in density) prior to 24 h of 37 °C culture through a humidified incubator containing 5% CO_2_ plus 95% O_2_.

Ginsenoside Rg1 (5, 10, and 20 µM as final concentrations) plus LPS (final concentrations of 0.1, 1, and 10 µg/mL for fibrosis induction) were applied to separately process BEND cells for 48 h. For the inhibition study, inhibitors including 5 mM N-acetylcysteine (NAC, ROS inhibitor; MedChem Express; Princeton, NJ, USA) and 10 μM MCC950 (NLRP3 inhibitor MCE; Princeton, NJ, USA) were incubated prior to treatment for 2 h, during which 1 µg/mL of LPS was added.

### 2.3. Cell Viability Assay

The viability of BEND cells was determined using a Cell Counting Kit-8 (CCK-8; MedChemExpress, HY-K0301) according to the manufacturer’s protocol. A total of 2 × 10^4^ cells were seeded into 96-well plates overnight and then exposed to 0.1, 1, and 10 µg/mL of LPS for 48 h. After removing the supernatant, the cells were incubated in 200 µL of a DMEM/F-12 medium containing a 20 µL CCK-8 solution for an additional 2 h at 37 °C and 5% CO_2_. The optical density (OD) values were measured at a wavelength of 450 nm.

### 2.4. Western Blotting Assay

A PMSF (1 mM)-containing ice-cold RIPA lysis buffer was applied to handle the cells washed using ice-cold PBS, and then Western blotting was implemented by reference to the methods in the previous literature. The primary antibodies against ASC (diluted at 1:1000; 10500-1-AP; Proteintech, Hoboken, NJ, USA) are N-cadherin (diluted at 1:1000; 22018-1-AP; Proteintech, Hoboken, NJ, USA), caspase-1 (1:1000 dilution ratio; 22915-1-AP; Proteintech, Hoboken, NJ, USA), α-SMA (diluted at 1:1000; 14395-1-AP; Proteintech, Hoboken, NJ, USA), E-cadherin (1:1000 dilution ratio; 20874-1-AP; Proteintech, Hoboken, NJ, USA), NLRP3 (diluted at 1:1000; 19771-1-AP; Proteintech, Hoboken, NJ, USA), β-actin (diluted at 1:3000; bs-0061R; Bioss, Beijing, China), and IL-1β (1:1000 dilution rate; bs-6319R; Bioss, Beijing, China). An HRP-labeled goat anti-rabbit antibody (dilution rate of 1:3000, bs-0295-HRP; Bioss, Beijing, China) was determined as the secondary antibody. Finally, the ECL solution was used for band examination, while ImageJ software (Version 1.38) was employed for signal quantification.

### 2.5. Determination of Mitochondrial ROS Level

DCFH-DA provided by the Beyotime Institute of Biotechnology (Shanghai, China) was used to detect the levels of total ROS. To be specific, BEND cells were first seeded into a 96-well microplate (5000 cells/well in density) for subsequent processing as per relevant instructions. Later, the cells were incubated (37 °C, 30 min) with 10 μmol/L DCFH-DA reagents away from light and rinsed in PBS gently three times. Subsequently, total ROS was examined by virtue of a Gemini XPS microplate offered by Reader Molecular Devices (Gothenburg, Sweden) for fluorescence intensity. Ultimately, a fluorescence microscope (Nikon, Tokyo, Japan) was employed to carry out microscopic analysis of the cell nuclei undergoing DAPI staining.

### 2.6. H&E and Masson’s Trichrome Staining

Tissue samples of mouse endometrium were fixed for 24 h with a 4% paraformaldehyde solution (0.04 g/mL). The fixed samples were rinsed under flowing water, dehydrated using graded alcohol, transparentized through xylene, and embedded in paraffin, followed by the preparation of 5 μm-thick paraffin sections for H&E staining and Masson staining. The dynamic histological changes of the mouse endometrium were appraised by means of an optical microscope (Olympus-DP73, Tokyo, Japan).

### 2.7. Evaluation of Oxidative Stress in Endometrium

The murine endometrium was assessed for oxidative stress by detecting the concentration of the lipid peroxidation marker malondialdehyde (MDA) together with the activity of the enzymatic antioxidants superoxide dismutase (SOD) and catalase (CAT) following the instructions of marketed kits for CAT (A007-1), MDA (A003-1), and SOD (A001-1) bought from Jiancheng Bioengineering Institute (Nanjing, China).

### 2.8. Data Presentation and Statistical Analyses

Statistical analyses were accomplished using SPSS 19.0 software (SPSS Inc., Chicago, IL, USA). All the data were tested for homoscedasticity in addition to normality, followed by the determination of significant differences by Duncan’s multiple range test combined with a one-way analysis of variance. The quantitative data were described in the format of mean ± SEM. *p* < 0.05 signified a difference of statistical significance.

## 3. Results

### 3.1. Ginsenoside Rg1 Inhibits LPS-Induced EMT Progression in BEND Cells

**Figure 1:**
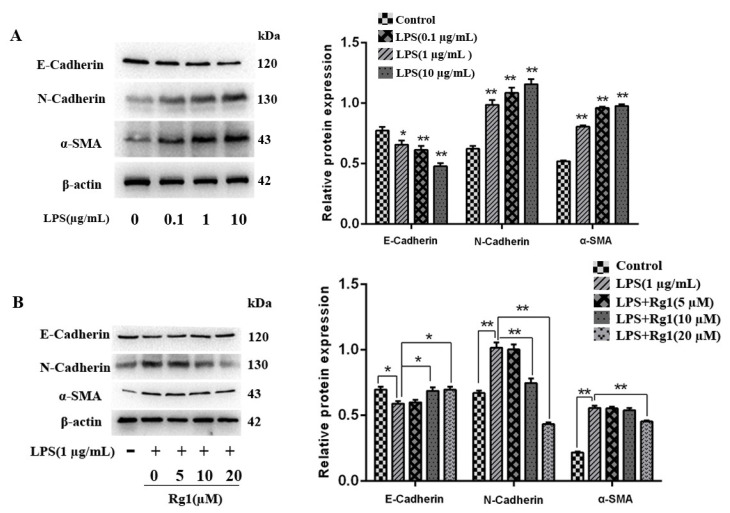
Ginsenoside Rg1 inhibits LPS-induced EMT progression in BEND cells. (**A**) BEND cells undergo 48 h of processing using LPS (0.1, 1, and 10 μg/mL). Proteins related to EMT (α-SMA, E-cadherin, and N-cadherin) (n = 3) were detected via Western blotting. (**B**) BEND cells are stimulated with LPS (1 μg/mL) and/or ginsenoside Rg1 at the concentrations required for 48 h. EMT-associated proteins N-cadherin, E-cadherin, and α-SMA (n = 3) were examined through Western blotting, with β-actin as the loading control. The mean ± SEM is adopted to express values; ** *p* < 0.01 and * *p* < 0.05 vs. the control group.

In order to determine the concentration of LPS-induced fibrosis in BEND cells, the fibrosis factors in groups treated with different concentrations of LPS were detected. The results of BEND cell viability is shown in Figure S1. E-cadherin expression declined as the LPS concentration increased (Figure 1A). Specifically, under the LPS concentration at 1 μg/mL, E-cadherin expression was significantly different from the control group (*p* < 0.01). By contrast with the control group, the LPS group had remarkably raised α-SMA plus N-cadherin expressions (dependent on the concentration) (*p* < 0.01). To validate whether ginsenoside Rg1 can alleviate LPS-induced fibrosis in BEND cells, the fibrosis-related factors in groups receiving ginsenoside Rg1 treatment at various concentrations were detected. E-cadherin expression was notably lower (*p* < 0.05), while N-cadherin and α-SMA expressions were evidently higher than the control group after 1 μg/mL of LPS treatment (*p* < 0.01) (Figure 1B). E-cadherin presented concentration-dependently elevated expression levels in ginsenoside Rg1 groups in comparison to those in the LPS group. There was a significant difference in E-cadherin between the LPS group and the group with a ginsenoside Rg1 concentration of 10 μM (*p* < 0.01). The ginsenoside Rg1 group displayed concentration-dependent decreases in N-cadherin and α-SMA expressions. Compared with the LPS group, the expression of N-cadherin varied markedly in the group treated with 10 μM ginsenoside Rg1 (*p* < 0.01), while an obvious difference in α-SMA expression in the LPS group was observed when the concentration of ginsenoside Rg1 was 20 μM.

### 3.2. Ginsenoside Rg1 Alleviates LPS-Induced EMT Progression in BEND Cells by Inhibiting ROS Production

**Figure 2:**
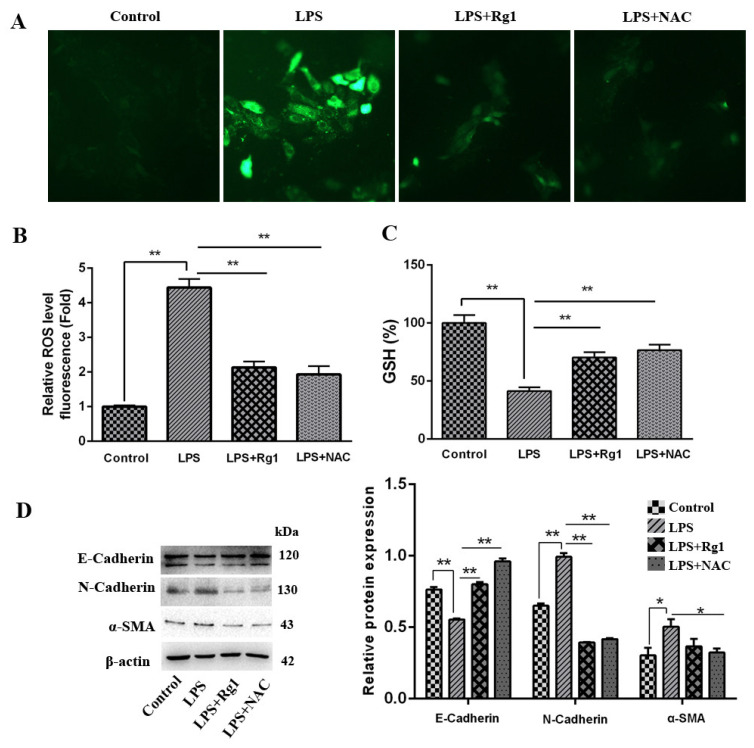
Ginsenoside Rg1 alleviates LPS-induced EMT progression in BEND cells by inhibiting ROS production. BEND cells are subjected to 1 μg/mL LPS, 20 μM ginsenoside Rg1, and/or 5 mM NAC processing for 48 h. (**A**,**B**) DCFH-DA for the detection of total ROS content (scale bar = 50 μm). (**C**) GSH level (n = 6). (**D**) Proteins E-cadherin, α-SMA, and N-cadherin associated with EMT (n = 3) were measured by Western blotting, with loading control set as β-actin. The mean ± SEM is selected as the expression for values; ** *p* < 0.01 and * *p* < 0.05 vs. the control group.

In order to verify whether ginsenoside Rg1 can reduce ROS generation in LPS-stimulated BEND cells, ROS staining and glutathione (GSH) were used to detect ROS fluorescence signals. The results in Figure 2A–C indicated that ROS was strongly positive in the LPS group compared with the control group, while it was weakly positive in groups treated with ginsenoside Rg1 and ROS inhibitor NAC (*p* < 0.01), and GSH content presented the opposite trends in those groups (*p* < 0.01). For the purpose of validating whether ginsenoside Rg1 alleviates LPS-induced fibrosis in BEND cells by inhibiting ROS production, fibrosis factors were detected. As shown in Figure 2D, by contrast to the LPS group, E-cadherin rose significantly in the ginsenoside Rg1 group and NAC group in terms of expression (*p* < 0.01). However, the ginsenoside Rg1 group and NAC group displayed apparently lowered N-cadherin and α-SMA expressions (*p* < 0.05).

### 3.3. Ginsenoside Rg1 Inhibits NLRP3 Activation by Reducing ROS in BEND Cells

**Figure 3:**
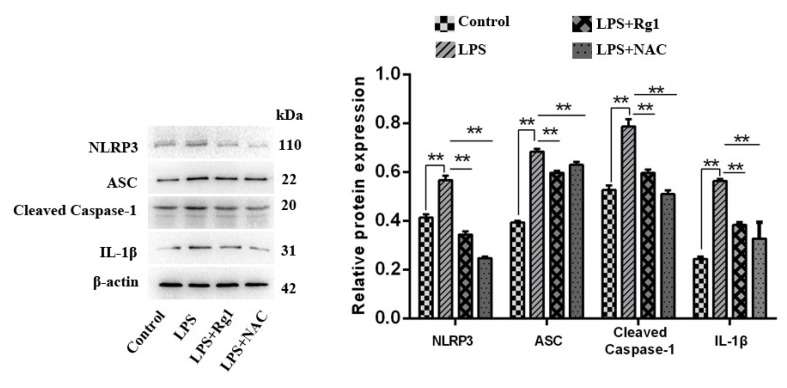
Ginsenoside Rg1 inhibits NLRP3 activation by reducing ROS in BEND cells. BEND cells undergo 48 h of 1 μg/mL LPS, NAC (5 mM), and/or ginsenoside Rg1 (20 μM) treatment. With β-actin as the loading control, Western blotting is conducted for the expression of NLRP3, ASC, cleaved caspase-1, and α-SMA proteins (n = 3). Values are described in the format of mean ± SEM; ** *p* < 0.01 vs. the control group.

For the purpose of validating whether ginsenoside Rg1 inhibits NLRP3 activation by reducing ROS in BEND cells, the expression of NLRP3, ASC, caspase-1, and IL-1β in different groups was detected. The LPS group had distinctly higher expressions of these factors than the control group (*p* < 0.01) (Figure 3). However, such factors significantly decreased in the ginsenoside Rg1 and NAC groups in comparison with those in the LPS group regarding expression (*p* < 0.01).

### 3.4. Ginsenoside Rg1 Alleviates BEND Cell EMT Progression by Inhibiting NLRP3

**Figure 4:**
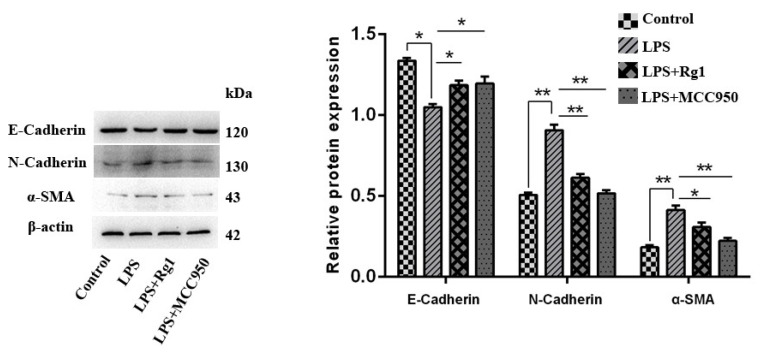
Ginsenoside Rg1 alleviates BEND cell EMT progression by inhibiting NLRP3. BEND cells undergo 48 h of treatment by means of 20 μM ginsenoside Rg1, 10 μM MCC950, and/or 1 μg/mL LPS. α-SMA, N-cadherin, and E-cadherin as EMT-related proteins were measured using Western blotting (n = 3), with the loading control from β-actin. The expression of values is mean ± SEM; ** *p* < 0.01 and * *p* < 0.05 vs. the control group.

In order to verify whether ginsenoside Rg1 alleviates the fibrosis of BEND cells by inhibiting NLRP3, fibrosis factors were detected. As shown in Figure 4, the ginsenoside Rg1 and MCC950 groups displayed prominently raised E-cadherin expression (*p* < 0.05) but substantially lowered N-cadherin, as well as α-SAM expression, compared to the LPS group (*p* < 0.05).

### 3.5. Ginsenoside Rg1 Alleviates Endometrial Fibrosis in Mice by Inhibiting ROS-Activated NLRP3

**Figure 5:**
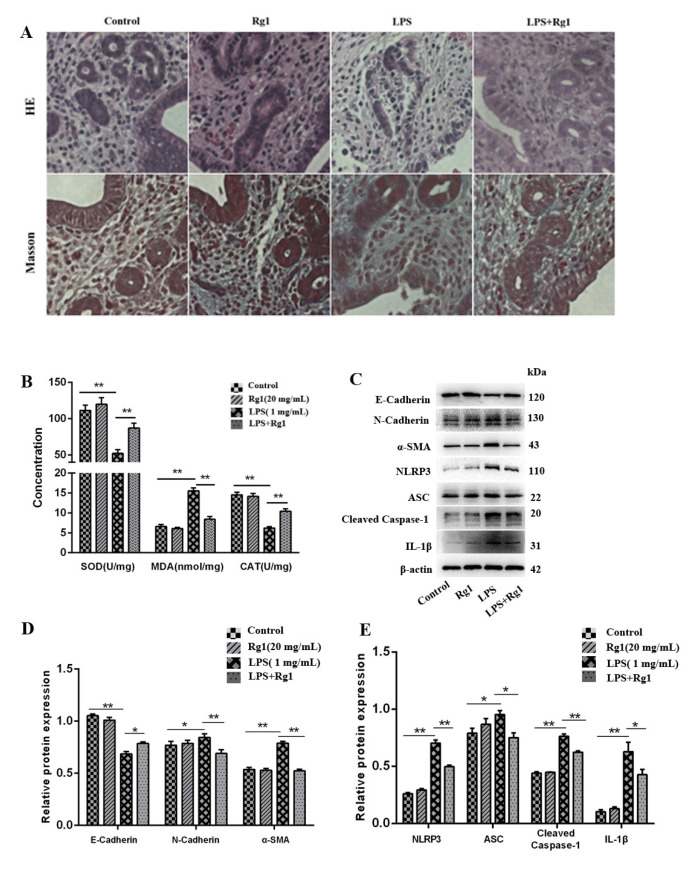
Ginsenoside Rg1 alleviates endometrial fibrosis in mice by inhibiting ROS-activated NLRP3. The control group (no treatment), the ginsenoside Rg1 group (100 mg/kg ginsenoside Rg1 treatment), the model group (with LPS), and the ginsenoside Rg1-treated group (with 100 mg/kg ginsenoside Rg1 + LPS) are set for the enrolled mice. (**A**) Uterine tissues undergoing H&E and Masson staining. (**B**) SOD, MDA, and CAT content obtained from uterine tissues. (**C**–**E**) α-SMA, E-cadherin, NLRP3, ASC, N-cadherin, caspase-1, and IL-1β (n = 3) were examined using Western blotting, for which the loading control was determined as β-actin. The mean ± SEM is adopted to describe values; ** *p* < 0.01 and * *p* < 0.05 vs. the control group.

Mice in this study were infused with LPS to induce endometritis and subsequently subjected to H&E and Masson staining. The normal endometrial structure was observed in both the control and ginsenoside Rg1 groups (Figure 5A). On the contrary, the structure of the endometrial basal layer in the endometritis group was incomplete, and the fibrous components were increased. The treatment combined with ginsenoside Rg1 could preferably protect the mice from endometritis triggered by LPS. Masson staining also showed mild collagen deposition and an EMT trend in the endometritis group, while the fibrosis rate was obviously reduced after ginsenoside Rg1 treatment (Figure 5A). The uterine CAT, MDA, and SOD content had no significant differences between the control and ginsenoside Rg1 groups. By comparing the LPS group with the control group, CAT and SOD significantly decreased, whereas a distinct increase in MDA was detected (*p* < 0.01). CAT and SOD also rose following ginsenoside Rg1 treatment, while the MDA content declined greatly in mice subjected to chronic D-gal injection (Figure 5B). A Western blotting assay (Figure 5C–E) further manifested that the LPS group possessed dramatically lower E-cadherin (*p* < 0.01) but overtly higher N-cadherin and α-SMA than the control group in terms of the expression levels (*p* < 0.05). After ginsenoside Rg1 treatment, an obvious elevation of E-cadherin expression (*p* < 0.05), together with apparent N-cadherin and α-SMA expressions (*p* < 0.01), decreased. Furthermore, caspase-1, NLRP3, IL-1β, and ASC exhibited significantly increased expressions in the LPS group in contrast with those in the control group (*p* < 0.05), while such expressions declined remarkably following ginsenoside Rg1 treatment (*p* < 0.05).

## 4. Discussion

Endometritis not only affects reproductive performance in addition to milk production in dairy cows but also leads to heavy economic loss [19]. As an important factor for organ fibrosis, the close relationship between inflammation and organ fibrosis has been confirmed by a large number of studies [20]. It has been reported that EMT mainly facilitates the pathogenesis of fibrosis [2], and it has been confirmed that an increase in α-SMA and N-cadherin, as well as a lack of E-cadherin, are biomarkers of EMT. For the purpose of alleviating endometrial fibrosis, the mechanism of inflammation-induced fibrosis needs to be elucidated. Given that *E. coli* is one of the main pathogens of cow endometritis [21], LPS was applied as the *E. coli* virulence factor to prepare an in vitro fibrosis model by stimulating BEND cells. In this experiment, it was uncovered that with an increase in LPS concentration, E-cadherin expression decreased, but α-SMA plus N-cadherin displayed increased expressions, indicating that LPS can induce the fibrosis of BEND cells.

The anti-fibrotic effect of Rg1 as a substance functioning against oxidation and inflammation in several organs, including the liver and kidney [10,11], has been confirmed. An in vitro LPS-induced fibrosis model was established using BEND cells in the present study. The results testified that treatment with Rg1 effectively reduced the mesenchymal markers, N-cadherin and α-SMA, but restored the epithelial marker, E-cadherin, from the aspect of the expression level. These findings are in line with previous studies demonstrating the potential of Rg1 to suppress the EMT process in hepatic stellate cells and rat renal tubular epithelial cells (NRK-52E) [16,17].

As revealed by prior investigations, excessive ROS-induced oxidative stress may result in chronic inflammation [4,5,6], which is an important characteristic of inflammatory response as well as a crucial factor for tissue and organ fibrosis [7,8]. As an antioxidant and anti-inflammatory substance, Rg1 can inhibit the ROS level in LPS-induced cardiomyocytes [22]. Meanwhile, Rg1 treatment is able to slow down renal fibrosis progression while postponing kidney aging by reducing ROS accumulation in the cortex of the kidney [23]. Similarly, in this experiment, it was observed that LPS could induce ROS accumulation in BEND cells, while Rg1 mitigated ROS generation and repressed the EMT process of BEND cells, similar to the effects of the ROS inhibitor NAC, indicating that Rg1 alleviates the LPS-induced fibrosis of BEND cells by inhibiting ROS production. In addition, given that ROS as a trigger of NLRP3, as a potent and selective inhibitor of NLRP3, MCC950 was used in this study [15,24,25], and our data showed that LPS promoted ASC, IL-1β, caspase-1, and NLRP3 at the expression level, which could be reduced by Rg1, similar to the effect of the NLRP3 inhibitor MCC950. These findings are consistent with the results obtained from renal fibrosis [26], pulmonary fibrosis [27], and myocardial fibrosis [28], suggesting that antioxidant therapy can reduce the activation of NLRP3 inflammasome, thereby alleviating fibrosis of tissues and organs.

Furthermore, whether Rg1 can significantly alleviate LPS-induced endometrial fibrosis in mice was explored in this experiment, thereby providing a possible basis for validating the effects on mitigating the EMT of endometrium in mice. Our data showed that LPS promoted endometrial fibrosis in mice, while Rg1 treatment restored the endometrium structure, which was consistent with the results concerning glomerular fibrosis in diabetic mice [29], suggesting that Rg1 can alleviate endometrial fibrosis in mice as well. Additionally, it was also revealed that following Rg1 processing, E-cadherin expression increased, and α-SMA together with N-cadherin had decreased expressions in LPS-induced murine endometrium, suggesting that Rg1 can reverse endometrial EMT. A recent study has shown that inflammation and EMT are key events contributing to organ fibrosis [20]. In diabetic rats, ginsenoside Rg1 is capable of markedly relieving podocyte EMT in addition to renal fibrosis [30]. By contrast to those in the control group in this study, ASC, caspase-1, IL-1β, and NLRP3 rose significantly in terms of expressions, and they declined subsequent to Rg1 treatment. These results are consistent with those observed in psoriatic skin injury, which showed that the NLRP3 inflammasome was repressed from activation, and inflammatory factors, including caspase-1, IL-1β, and ASC, presented lowered levels in mice, suggesting that Rg1 can repair the psoriasis-like skin injury by down-regulating NLRP3 inflammatory factors [31]. Therefore, our results suggest that Rg1 exerts a vital anti-inflammatory effect to reverse endometrial fibrosis in mice.

## 5. Conclusions

To sum up, the experimental data in this study indicate that ginsenoside Rg1 is able to alleviate endometrial fibrosis induced by the LPS of BEND cells, which is related to the inhibition of ginsenoside Rg1 on ROS accumulation, thereby restraining NLRP3 inflammatory factors from expression. The experiments on mice showed that ginsenoside Rg1 could not only reduce NLRP3 inflammatory factors in addition to the EMT process in murine endometrium but also alleviate endometrial fibrosis in mice. The aforementioned results can serve as powerful preclinical evidence for the potential of ginsenoside Rg1 to become an alternative drug for curing endometrial fibrosis.

## Data Availability

The datasets used and/or analyzed in the current study are available from the corresponding author upon reasonable request.

## Supplementary Materials

The following supporting information can be downloaded at: https://www.mdpi.com/article/10.3390/ani13233723/s1, Figure S1: The viability of BEND cells. BEND cells undergo 48 h of processing using LPS (0.1, 1 and 10 μg/mL), the viability of BEND cells was determined using a CCK-8, the optical density (OD) values were measured at a wavelength of 450 nm. The mean ± SEM is adopted to express values; * *p* < 0.05 vs. control group.
