# Supplementary material for: Ginsenoside Rg1 Alleviates Lipopolysaccharide-Induced Fibrosis of Endometrial Epithelial Cells in Dairy Cows by Inhibiting Reactive Oxygen Species-Activated NLRP3
Source: Animals (Basel). 2023 Dec 1;13(23):3723. doi: 10.3390/ani13233723 (PMC10705063; doi:10.3390/ani13233723)
Supplement: Supplementary file 1 [file animals-13-03723-s001.zip › animals-2715977-supplementary.pdf]

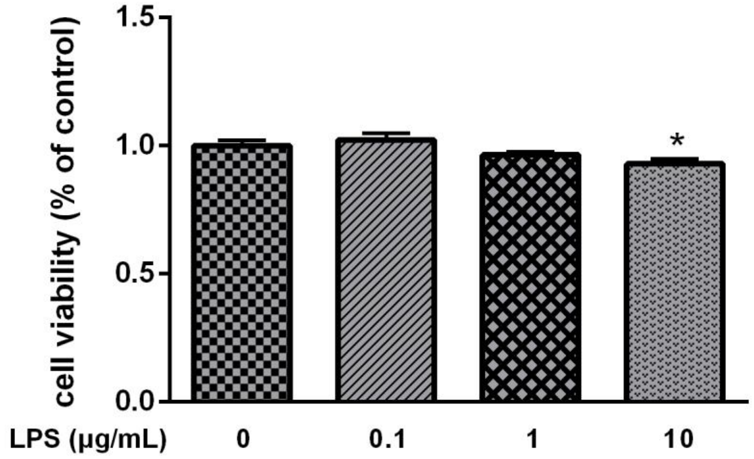

Figure S1. The viability of BEND cells. BEND cells undergo 48 h of processing using LPS (0.1, 1 and 10 µg/mL), the viability of BEND cells was determined using a CCK-8, the optical density (OD) values were measured at a wavelength of 450 nm. The mean ± SEM is adopted to express values; * $p < 0.05$ vs. control group.
